# Study of spontaneous mutations in the transmission of poplar chloroplast genomes from mother to offspring

**DOI:** 10.1186/s12864-018-4813-8

**Published:** 2018-05-29

**Authors:** Sheng Zhu, Meng Xu, Haoran Wang, Huixin Pan, Guangping Wang, Minren Huang

**Affiliations:** Co-Innovation Center for Sustainable Forestry in Southern China, Nanjing Forestry University, Nanjing, 210037 China

**Keywords:** Chloroplast genome, *Populus*, Transmission, Multi-pass sequencing, Full-sibling family

## Abstract

**Background:**

Chloroplasts have their own genomes, independent from nuclear genomes, that play vital roles in growth, which is a major targeted trait for genetic improvement in *Populus*. Angiosperm chloroplast genomes are maternally inherited, but the chloroplast’ variation pattern of poplar at the single-base level during the transmission from mother to offspring remains unknown.

**Results:**

Here, we constructed high-quality and almost complete chloroplast genomes for three poplar clones, ‘NL895’ and its parents, ‘I69’ and ‘I45’, from the short-read datasets using multi-pass sequencing (15–16 times per clone) and ultra-high coverage (at least 8500× per clone), with the four-step strategy of Simulation–Assembly–Merging–Correction. Each of the three resulting chloroplast assemblies contained contigs covering > 99% of *Populus trichocarpa* chloroplast DNA as a reference. A total of 401 variant loci were identified by a hybrid strategy of genome comparison-based and mapping-based single nucleotide polymorphism calling. The genotypes of 94 variant loci were different among the three poplar clones. However, only 1 of the 94 loci was a missense mutation, which was located in the exon region of *rpoC1* encoding the β’ subunit of plastid-encoded RNA polymerase. The genotype of the loci in NL895 and its female parent (I69) was different from that of its male parent (I45).

**Conclusions:**

This research provides resources for further chloroplast genomic studies of a F_1_ full-sibling family derived from a cross between I69 and I45, and will improve the application of chloroplast genomic information in modern *Populus* breeding programs.

**Electronic supplementary material:**

The online version of this article (10.1186/s12864-018-4813-8) contains supplementary material, which is available to authorized users.

## Background

Poplar is an important plantation tree species being genetically improved. The genetic improvement of traits associated with growth is a major facet of *Populus* breeding [1]. Chloroplasts are important organelles that supply energy for the growth and development of green plants through photosynthesis. Chloroplast genomes contain many genes associated with photosynthesis, such as genes encoding ribulose-(1,5)-bisphosphate carboxylase/oxygenase (RuBisCO) [2] and plastid-encoded RNA polymerase (PEP) [3].

High-quality chloroplast genomes are essential for the genome comparison of closely related organisms, such as *Populus* full- or half-sibling (sib) progeny. The patterns of genetic variation in chloroplast DNAs (cpDNAs) among full-sib progeny and their parents, which can be identified through the comparison of whole-chloroplast genome sequences, contain valuable information for hybrid tree breeding. The maternal inheritance of cpDNAs in the genus *Populus* was determined by restriction fragment analysis [4]. However, it is very difficult to study the uniparental inheritance mode of cpDNAs within a *Populus* full-sib family using only that method due to the ultra-high level of cpDNA sequence similarities and the limited restriction enzyme sites in the chloroplast genome [5, 6]. The transmission of cpDNAs from the maternal parent to F_1_ offspring may be analyzed at both the whole-chloroplast genome and single-base levels, using a whole-chloroplast genome comparison. In addition, the effects of a cross between *Populus deltoides* as the maternal parent and *Populus × euramericana* as the paternal parent is usually superior to those of its reciprocal cross [7]. For instance, Wang and Huang et al. selected and obtained a few superior clones, such as Nanlin895 (NL895) (http://www.shtree.com/nl-895.htm) and NL95 (http://www.shtree.com/nl-95.htm), from the F_1_ hybrids between *P. deltoides* Bartr. cv. ‘I-69/55’ (I69, ♀) and *P. × euramericana* Guinier. cv. ‘I-45/51’ (I45, ♂). The differences between the reciprocal crosses is difficult to explain using Mendelian inheritance modes, such as the biparental inheritance of the nuclear genome, but it can be explained by the uniparental inheritance of cpDNA. However, determining the genetic mechanism underlying the differences between reciprocal crosses is beneficial for parental selection as an important part of *Populus* breeding programs. Thus, the comparison of high-quality chloroplast genomes has the potential to provide chloroplast genome-associated information for the genetic improvement of *Populus* through the use of hybrid breeding technology.

Complete chloroplast genome construction consists of two parts: obtaining chloroplast reads and constructing chloroplast genome sequences from the reads. In general, to generate cpDNA reads, cpDNA sequences are isolated from whole cells or extracted from whole-genome shotgun reads. Because of the advances in next-generation sequencing (NGS), in terms of time and cost, and the increases in the number of available chloroplast genomes, whole-genome shotgun sequencing based on NGS technology is increasingly used to construct plant chloroplast genomes, such as *Brassica rapa* and *Raphanus sativus* [8, 9].

The chloroplast genome sequences of six poplar species, belonging to four sections in the genus *Populus*, are available from the NCBI Organelle Genome Resources (http://www.ncbi.nlm.nih.gov/genome/organelle/). Although poplar chloroplasts have conserved plastid genomes of < 160 kilobase pairs (kbp) in length, the de novo assembly of complete chloroplast genomes for *Populus* species remains challenging because of a pair of inverted repeats, IRa and IRb, of approximately 27 kbp in size and more than 99.5% sequence similarity (as described in the Results section). This study aims to construct high-quality poplar chloroplast genomes from whole-genome shotgun Illumina reads using de novo and reference-guided strategies.

Here, we obtained chloroplast HiSeq 2000 reads at a total sequencing depth of > 8500× each extracted from the whole-genome shotgun and multiple passes (15–16 times per clone) sequencing for three poplar clones, including NL895 and its parents (I69 and I45). The high-quality chloroplast genomes of the three poplar clones were constructed by a combination of eight different de novo assemblers and the assemblies merged tool CISA [10]. Whole-chloroplast genome comparisons among the three clones were performed to analyze the genetic variations occurring during the transmission process of cpDNAs from mother to offspring.

## Results

### Feasibility evaluation of the reference-assisted strategy

In this study, we used the hybrid strategy of both reference-assisted and de novo assembly to isolate and construct a complete chloroplast genome. Evaluating the applicability of the reference-assisted strategy to the *Populus* cpDNAs’ assembly is required before being used. The feasibility of the reference-assisted strategy was assessed on the basis of several aspects, including the degree of chloroplast sequence similarity between the target and reference species, the de novo assembly of short-read data simulated from the reference chloroplast genome, and the proportion of the chloroplast reads in all of the reads generated from whole-cell sequencing.

#### Sequence similarities of *Populus* cpDNAs

**Fig. 1:**
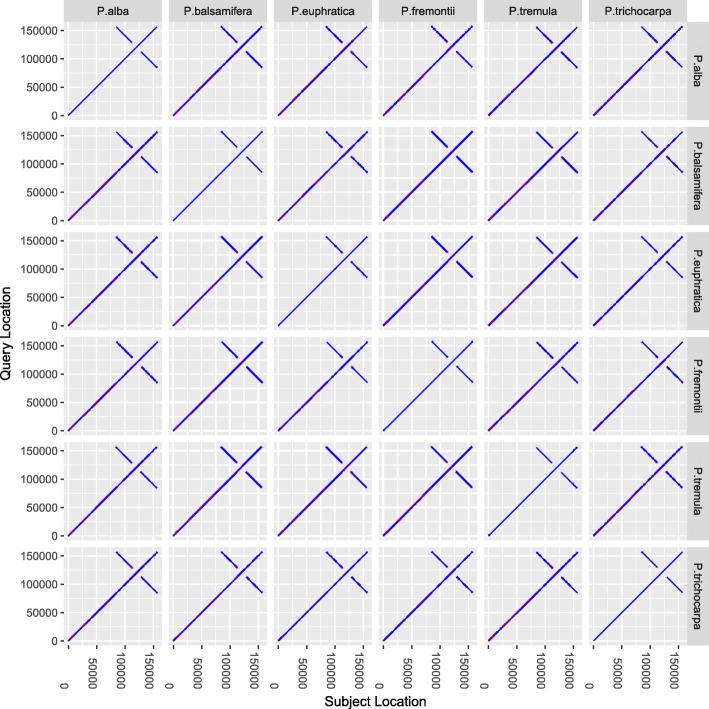
Whole-genome dot-plot comparison of six *Populus* cpDNAs. The chloroplast genomes of all six *Populus* species, *P. alba*, *P. tremula*, *P. euphratica*, *P. fremontii*, *P. balsamifera* and *P. trichocarpa*, were compared using LASTZ (v1.03.28). The aligned blocks are represented as blue lines. The blocks aligned in the reverse orientation are a pair of inverted repeats (IRa & IRb) in the chloroplast genomes. The starts and ends of the aligned blocks are labeled with transparent red points

The highly conserved features of the chloroplast genomes are well-known. However, it was unclear whether the reference-assisted method was suitable for constructing the *Populus* chloroplast genome from whole-genome sequencing reads, owing to the unknown levels of sequence similarity among cpDNAs from species in the genus *Populus* L. Thus, we performed pairwise comparisons among the cpDNA sequences from six *Populus* species, *P. alba*, *P. tremula*, *P. euphratica*, *P. fremontii*, *P. balsamifera* and *P. trichocarpa*, using the LASTZ genome alignment tool (Fig. 1). The 156–157-kbp chloroplast genomes from the six *Populus* species, belonging to four *Populus* sections, had an over 99.5% sequence identity. These *Populus* chloroplast genomes shared the same quadripartite structure, including an 84–85 kbp large single-copy region (LSC) and a 16-kbp small single-copy region (SSC) separated by a pair of inverted repeats (IRs), IRa and IRb, with sequence lengths of 27 kbp and a sequence similarity of > 99.5%. Compared with the other five *Populus* species, the *P. trichocarpa* chloroplast genome had a relatively higher sequence similarity (~ 100%) between IRa and IRb.

The complete chloroplast genomes of these species within the genus *Populus* exhibited relatively high levels of sequence similarity. However, it was still unknown whether all chloroplast reads can be extracted from whole-genome shotgun sequencing reads generated on an Illumina sequencing platform using the reference-assisted strategy. The Illumina-like and error-free reads, which were obtained from six *Populus* cpDNAs using the split-read method (as described in the ‘Materials and Methods’ section), were mapped to each cpDNA of these *Populus* species using Bowtie and BWA (Additional file 1: Table S1). The average percentage of short reads mapped to *P. trichocarpa* cpDNA (95%) as a reference was slightly higher than those of the other five *Populus* cpDNAs as references. A total of 95.8% reads, which were generated from the *P. fremontii* (*Populus* Sect. Aigeiros) chloroplast genome through the split-read method, were mapped to the *P. trichocarpa* cpDNA. Based on the metrics of both sequence similarity and the mapping ratio, the *P. trichocarpa* chloroplast genome was selected as a reference for subsequent analyses.

#### Assembly of the simulated reads

The cost of DNA sequencing is frequently a consideration in de novo chloroplast genome assembly projects. To calculate the effective cost of whole-genome shotgun sequencing, it is necessary to estimate the amounts of short reads required for the generation of a high-quality assembly of the chloroplast genome. The minimum amount of chloroplast reads for de novo assembly and the ratio of chloroplast reads to whole-genome sequencing reads are two basic elements for estimating the required number and cost of whole-genome shotgun reads.

The numbers and lengths of chloroplast reads are two key factors for constructing chloroplast genomes using the shotgun sequencing strategy. To assess the effects of the two factors on the chloroplast genome assembly, 12 short-read datasets for all combinations of the three different read lengths (60, 80 and 100 bp) with four different read amounts—10 k (10^4^), 100 k (10^5^), 1 M (10^6^) and 10 M (10^7^) of paired-end reads—were simulated with *P. trichocarpa* cpDNA as a reference and used for the simulation of de novo poplar chloroplast genome assembly.

First, we used KmerGenie to estimate the genome sizes for the 12 combinations of read lengths and read amounts (60 bp–10 k, 60 bp–100 k, 60 bp–1 M, 60 bp–10 M, 80 bp–10 k, 80 bp–100 k, 80 bp–1 M, 80 bp–10 M, 100 bp–10 k, 100 bp–100 k, 100 bp–1 M and 100 bp–10 M). The estimated values of four datasets, including 60 bp–100 k, 80 bp–100 k, 100 bp–100 k and 60 bp–1 M, were the nearest to the genome size of the reference cpDNA (*P. trichocarpa*), and ranged from 129.7 kbp (82.6%) to 130.7 kbp (82.8%) (Additional file 1: Table S2). The estimated size appeared equal to the total size of the combined LSC, IRa and SSC regions, rather than the combined size of all four regions, LSC, IRa, SSC and IRb, of the *Populus* cpDNA (157 kb). This may be due to the extremely high level of sequence similarity (~ 100%) between the IRa and IRb regions, which are 27 kbp in length.

The de novo assembly of each of the 12 read datasets was performed using Minia with five k-mer values of 19, 29, 39, 49 and 59. A total of sixty (12 datasets × 5 k-mers) assemblies were obtained and used for estimating the effects of the three factors (read length, read amount and k-mer value) on chloroplast genome assembly.

**Fig. 2:**
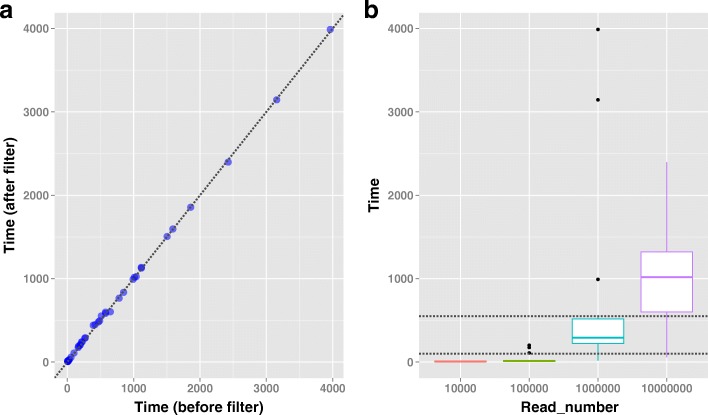
Running times for assembling the simulated short reads data. **a** The running times for assembling the simulated short reads data before or after filtering. The *x*-axis and *y*-axis represent the assembly times (in seconds) of the simulated short reads before and after which PCR duplicates were filtered from raw data, respectively. The times for assembling the simulated short reads data before or after filtering are indicated by the transparent blue circles. The equation ‘*y* = *x*’ is plotted as the black dotted line. **b** Boxplot of the running times for assembling four simulated reads data sets (10^4^, 10^5^, 10^6^ and 10^7^ pairs of short reads). The assembly times for 10^4^, 10^5^, 10^6^ and 10^7^ pairs of simulated reads are colored as red, green, cyan and purple, respectively. Two black dashed lines in the figure represent two equations ‘*y* = 550’ and ‘*y* = 100’, respectively

Time consumption and the quality of the de novo genome assembly are the two most important aspects of the cpDNA assembly. The time required for these 60 assemblies was not significantly different between before and after the PCR duplicate removal (Fig. 2a), because there were < 0.1% PCR duplicates. The effects of k-mer, read length and read amount on the assembly time were tested using an analysis of variance (ANOVA). There was a statistically significant difference (*P* = 4.58e^− 5^) in the assembly times among all four levels of read amounts (10 k, 100 k, 1 M and 10 M). To determine which level of read amount was different from the others, pairwise comparisons of the assembly time among the four read amounts were performed using Tukey’s multiple comparison. The running times for the 10-k and 100-k pair-read datasets were significantly different from those of the 1-M and 10-M pair reads (Fig. 2b). In addition, the effects of the pairwise interactions between k-mer value, read length and read amount were not detected by two-way ANOVA.

**Fig. 3:**
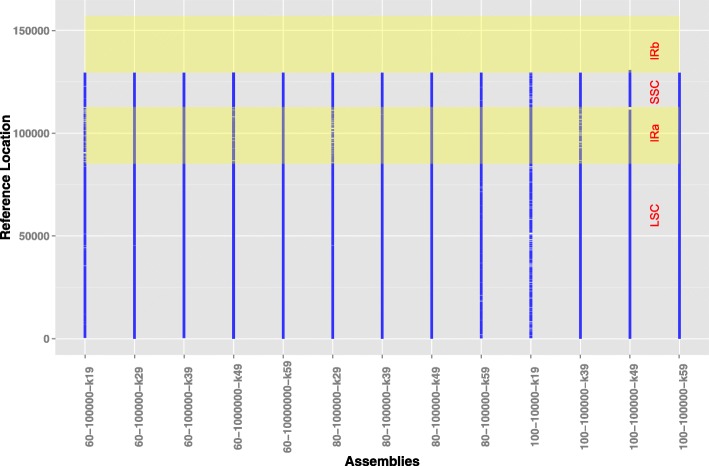
The fraction of *P. trichocarpa* chloroplast genome covered by the assemblies of the simulated short reads under multiple k-mer values. The resulting assemblies with genome fractions > 128 kbp are shown in this figure. The quadrant structure of the chloroplast genome is composed of large single-copy (LSC) and small single-copy (SSC) regions separated by a pair of inverted repeats (IRa and IRb); the regions of both IRa and IRb are labeled with the wide transparent yellow band. The *x*-axis and *y*-axis represent the genome assemblies for the simulated reads data and the locations of the reference genome covered by contigs from the genome assemblies, respectively

Like the assembly time, the resulting 60 assemblies were not different between the before and after PCR duplicate removal in terms of the frequently-used assembly metrics, such as total length, contig number, mean length, min length, max length, N80/L80, N50/L50 and N20/L20 (N80/L80, N50/L50 and N20/L20 are defined by QUAST, http://quast.bioinf.spbau.ru/manual.html). To further compare the qualities of the 60 assemblies on 12 simulated read datasets, we selected the assemblies having total bases of > 80% the reference genome size (157 kbp) and resulting contigs of at least 200 bp in length. A total of the 13 assemblies from 6 simulated read datasets, including 60 bp (read length)–100 k (read pairs), 60 bp–1 M, 60 bp–10 M, 80 bp–100 k, 100 bp–10 k and 100 bp–100 k, were selected and used for further analyses (Additional file 1: Table S3). However, a large proportion of contigs for each of the 13 assemblies were far < 1 kbp in length. To decrease the contig numbers and increase assembly contiguity, the resulting contigs were merged into the larger contigs using the reference chloroplast genome as a guide. After merging contigs in the 13 assemblies, the number of contigs was greatly reduced, with only one contig in the three assemblies. The genome fraction rates of the 13 assemblies after contig merging ranged from 73.56 to 82.40% and were close to the estimated genome size as determined by KmerGenie. The merged contigs for these assemblies occupied the almost complete LSC, IRa and SSC regions on the reference cpDNA (Fig. 3). The IRb region was missing in the resulting assemblies because of the nearly 100% sequence identity between IRa and IRb on the *P. trichocarpa* cpDNA. The three assemblies consisting of only one contig were assembled from the 100-k pair-read datasets with 60-, 80- and 100-bp read lengths, and their genome coverage rates ranged from 82.20 to 82.40%. In addition, the average number of mismatches per 100 kbp of aligned bases for the 13 assemblies ranged from 3.16 to 10.05.

Based on the metrics used for assessing the chloroplast assemblies on these 12 simulated datasets, the number of reads seemed to be a more important factor than read length. The most complete three assemblies were obtained from three datasets of 100-k pair reads, and the genome sizes of these assemblies were approximately equal to the estimated genome size. Thus, we presumed that 100 k–1 M pairs may be the suitable number of reads required for constructing the *Populus* chloroplast genome.

#### Proportion of the chloroplast reads

The ratio of cpDNA reads to whole-genome sequencing reads is another key factor that contributes to the estimation of the amount of whole-genome shotgun reads required for the construction of the *Populus* chloroplast genome. The chloroplast reads were isolated from all of the whole-genome short reads for the I69, I45 and NL895 clones, by aligning reads against the *P. trichocarpa* cpDNA using BWA and Bowtie aligners. The cpDNA ratios of read datasets for the three poplar clones I45, I69 and NL895 were ~ 4.2, 7.5 and 5.0%, respectively (Additional file 1: Table S4). The ratio of cpDNA reads for each poplar clone was not significantly different among multiple datasets (15–16), except the first dataset.

**Fig. 4:**
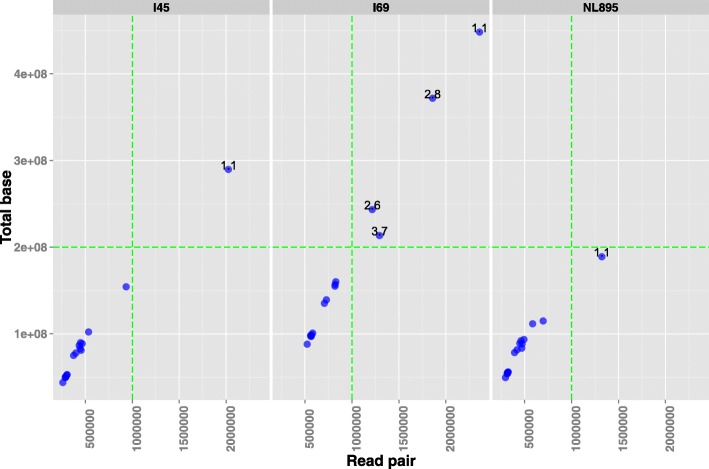
The total base count and read pairs of 48 short-read datasets. The *x*-axis and *y*-axis represent the total base count and the read pairs (read amount) of 48 short-read datasets from three poplar clones (I45, I69 and NL895), respectively. The horizontal and vertical green dashed lines represent the equations ‘*y* = 2 × 10^8^’ and ‘*x* =1 × 10^6^’, respectively

Each of the 47 real datasets of whole-genome shotgun sequencing had chloroplast reads of more than 10^5^ (100 k) pairs. Of these, 41 datasets contained chloroplast reads ranging from 100 k to 10^6^ (1 M) pairs, which was similar to the amount of whole-genome shotgun reads required for cpDNA assembly in the simulated reads study above. More than 1 M pairs of chloroplast reads were extracted from the six short-read datasets, including one dataset for the clone I45, four datasets for I69 and one dataset for NL895 (Fig. 4). The numbers of chloroplast reads from the poplar clones I45 and NL895 were less than those of clone I69.

### Chloroplast genome assembly

We employed four different parameter strategies to assemble a chloroplast genome from each of the 47 chloroplast read datasets, which were isolated from whole-genome sequencing reads from the three poplar clones I45, I69 and NL895. The first strategy used multiple k-mer values (k = 19, 21, 23…63) with which chloroplast genomes were de novo assembled using four assembly tools (ABySS, Minia, SOAPdenovo2 and Velvet). The second strategy ran the assembler SGA using multiple overlap sizes (m = 41, 45, 51, 55, 61 and 63). The third strategy ran the assembler IDBA using multiple differential iterative steps (step = 2, 4, 8, 10, 20 and 30). The last strategy ran the SPAdes and Edena assemblers using default parameters. A total of 4982 assemblies were performed on all 47 datasets using the eight assembly tools with the corresponding parameter strategies, 4980 of which successfully obtained contigs.

#### The estimated genome size

The cpDNA genome sizes of the three poplar clones (I45, I69 and NL895) were estimated from the all 47 datasets based on the k-mer distribution before the de novo assembly of the poplar chloroplast genome. The genome size estimates of all 47 datasets were very close to 129 kbp, which was approximately equal to the total bases of the LSC, IRa and SSC regions of the *Populus* chloroplast genome. This was in accordance with the results of the previously performed simulated reads analysis. Nevertheless, the estimated values of read dataset (Set) 1.1 of both clones I45 and NL895 were far less than 129 kbp. This may be because the read amounts of these two datasets were greater than 1 M (10^6^) pairs, which was far from the number of reads required for the poplar chloroplast genome’s assembly.

#### The time used for cpDNA assembly

De novo short-read assembly requires intensive computational time. The running time for de novo cpDNA assembly may be affected by a number of factors, such as data volume (total numbers or total bases of short reads), assembly tools and assembly parameters. There seems to be a positive correlation between data volume and the running time. The correlation coefficient values for the total number of reads and their running times using all eight assemblers ranged from 0.70 to 0.98, and were slightly lower than those for total bases and their running times. The running times using the same assembler generally increased with the increasing amounts of short reads used for cpDNA assembly, which seemed to also be consistent with the results of the previous simulated read assembly.

Obviously, the assembly times for the same datasets were different among the eight assemblers. For example, the assembly times using SPAdes of all 47 read datasets were more than those using Edena. In comparison with the assemblies of SPAdes and Edena that were performed under the default parameters only, the assemblies of SGA, IDBA, ABySS, Minia, SOAPdenovo and Velvet on the same dataset were performed using several customized parameters. The running times of assemblies generated by the six assemblers may be influenced not only by read volume and assembly tools, but also by different parameters. To determine the effects of both read volume and parameters on the running time, we used a multivariate ANOVA to analyze each of the assemblies generated by the six assemblers. The running times of the six assemblers (*p* < 0.001), except SGA (*p* = 0.07), were significantly different for multiple values of the corresponding parameters. The running times of the IDBA assemblies on the same read dataset increased in general with the decreasing ‘step’ parameter as a result of the increased number of iterations in an assembly when the ‘step’ parameter was set to the smallest value. The assembly times of the four k-mer-based assemblers (ABySS, Minia, SOAPdenovo and Velvet) increased broadly with the decreasing k-mer values. Thus, the running times of short-read assemblies for constructing chloroplast genomes were mainly under the significant influence of the three factors, including data (reads) volume, assembly tools and assembly parameters. Additionally, the running times for the SGA were longer than those of the other seven assemblers, based on the results from multiple comparison tests.

#### The initial de novo assembly

From the 4980 resulting assemblies, we screened 2200 assemblies having total base lengths of > 126 kbp and total contig numbers of < 100, which were selected as a preliminary selection criteria on the basis of the previous simulated reads analysis. The number of read datasets that were assembled and met the selection criteria are summarized in Table 1. ABySS, IDBA and SPAdes were the only three assembly tools that successfully assembled the 47 datasets into eligible assemblies. SGA was the least successful assembler, producing eligible assemblies from only 2 of the 47 datasets.

**Table 1:** The number of read datasets successfully assembled by each assembly tool

Clone	Assembly tool							
	ABySS	Edena	IDBA	Minia	SGA	SOAPdenovo	SPAdes	Velvet
I45	16	5	16	16	0	6	16	8
I69	15	1	15	12	0	3	15	7
NL895	16	3	16	15	2	6	16	6

**Fig. 5:**
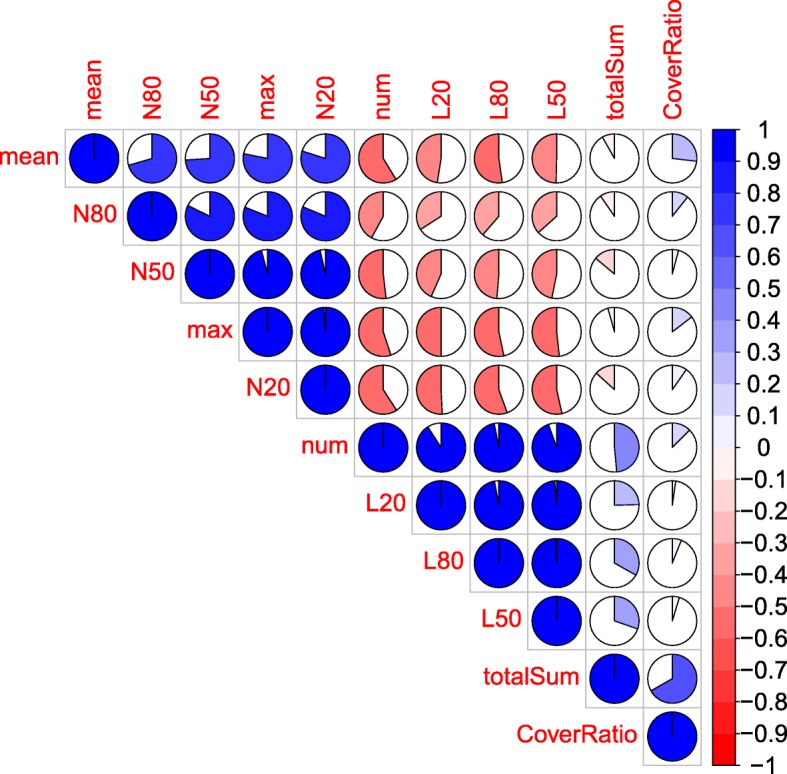
The correlations among 11 metrics for a genome assembly assessment. This figure, which was plotted using the R package ‘corrplot’, represents the correlation matrix of the 11 metrics used for assessing poplar chloroplast genome assembly. The 11 metrics in the figure were ‘num’ (number of contigs), ‘totalSum’ (the total base of contigs), ‘mean’ (the mean length of contigs), ‘max’ (the max length of contigs), ‘CoverRatio’ (the ratio of reference chloroplast genome covered by the aligned contigs), N80, L80, N50, L50, N20 and L20. N80, L80, N50, L50, N20 and L20 are defined by QUAST

To effectively select the optimum assembly from each assembler using each dataset, we analyzed the relationships among 11 assembly metrics, including the numbers of contigs (num), the total bases of contigs (totalSum), the mean lengths of contigs (mean), the max lengths of contigs (max), N80 s and L80 s of contigs (N80 and L80), N50 s and L50 s of contigs (N50 and L50), N20 s and L20 s of contigs (N20 and L20), and the ratio of the reference chloroplast genome covered by the aligned contigs (CoverRatio). The relationships among the 11 metrics are shown in Fig. 5. The correlation coefficients between N50 and four metrics, N80, N20, max and mean, were at least 0.74. The coefficients between num and three metrics, L80, L50 and L20, had high values of > 0.91. totalSum and CoverRatio had very low correlations with the other nine metrics. Thus, four metrics, N50, num, totalSum and CoverRatio, were used to select the optimum assembly from each assembler using each dataset under differential parameters, such as k-mer, overlap and iterative step. For assemblies from the same assembler using the same datasets, there were no differences in the num metric among the different parameters. For the assemblies of the IDBA, Minia, SOAPdenovo, SGA and Velvet five assemblers, no significant differences were shown in the CoverRatio and totalSum among the different parameters. However, the CoverRatio and totalSum of the ABySS assemblies with certain k-mers were relatively higher than those of the other k-mers. Thus, the selection criteria for the optimum assemblies for the ABySS, IDBA, Minia, SOAPdenovo, SGA and Velvet assemblers were as follows:

- Of the assemblies generated by IDBA, Minia, SOAPdenovo, SGA and Velvet assemblers, the assemblies with the greatest N50 values were selected from the assemblies using the same dataset under different parameters as the optimum cpDNA assembly.
- For the ABySS assemblies, the assemblies with the greatest N50 contig sizes were selected as the optimum when the CoverRatio for the same dataset was less than 85%. Otherwise, we chose the assembly with the greatest N50 from ABySS assemblies with CoverRatios of > 85% as the optimum assembly.

Based on the selection criteria, we obtained 235 optimum assemblies, 2 assemblies from SGA, 9 from Edena, 15 from SOAPdenovo, 25 from Velvet, 43 from Minia and 47 from the three assemblers ABySS, IDBA and SPAdes. To further analyze these assemblies, we compared the N50, num, totalSum and CoverRatio metrics. The N50 values of the Edena, Minia and Velvet assemblies were far less than those of the other five tools. The num (contig number) values of the Edena, Minia and Velvet assemblies were relatively high at greater than 42 compared with those of the other five assemblers (less than 25). The totalSum (total base) values of the Velvet assemblies were much greater than those of the other tools, being at least 225 kbp, which greatly deviated from the 157 kbp of the *Populus* reference chloroplast genome. The CoverRatio values of the SGA and SOAPdenovo assemblies were less than 80.6%. Thus, the Edena, Minia, SGA, SOAPdenovo and Velvet assemblies were inferior to those of the remaining three assemblers, ABySS, IDBA and SPAdes. Thus, the ABySS, IDBA and SPAdes assemblies were used for the following improved assembly.

#### The improved assembly

The IR sequence was easily lost in the de novo assembly of the entire *Populus* chloroplast genome owing to the high similarity between IRa and IRb. The genome size estimates of the simulated data and real data in the study were approximately 129 kbp, covering the LSC + IRa + SSC regions, and almost equal to those of the de novo assemblies. Thus, we divided the entire chloroplast assembly into two parts, the LSC + IRa + SSC and the IRb regions.

However, there were no significant differences among the SPAdes, IDBA and ABySS assemblies of each dataset for the I45, I69 and NL895 poplar clones. Thus, it is very difficult to select the optimum assembly from those generated by the SPAdes, IDBA and ABySS assemblers for each dataset. To further improve the quality levels of the cpDNA assemblies from the three poplar clones, we adopted the strategy of merging multiple assemblies into one to reduce errors and to extend contig lengths. Given the preferable contiguity of the assemblies generated by SPAdes relative to those of the other two assemblers, the SPAdes assemblies were selected as input for merging the initial contigs. The contigs from all of the SPAdes assemblies for each poplar clone were merged into large contigs using the genomic assemblies merging tool CISA(v1.3) after the removal of misassembled contigs identified by QUAST (v3.1). By merging these contigs based on the MUMmer (v3.23) pairwise alignment, the large contigs were merged into three super contigs of 128,701, 128,864 and 128,786 bp for the three clones I45, I69 and NL895, respectively. All three super contigs were aligned to 1–129,435 bp of the *P. trichocarpa* chloroplast genome, fully covering the LSC + IRa + SSC region.

The two chloroplast IRs, which are long equal blocks of ultra-high sequence similarity, posed a challenge for constructing the high-quality assembly of chloroplast genome sequences. The acquisition of the contigs spanning the IRb region was undertaken in the three steps. Frist, contigs that overlapped with IRb were selected from all of the assemblies generated by the SPAdes, IDBA and ABySS assemblers on 15 or 16 datasets per poplar clone. Next, these selected contigs were integrated into the contigs completely containing or partly overlapping the IRb region, on the basis of its genomic comparison with the reference cpDNA. We obtained three IRb-overlapped contigs of 30,817, 30,819 and 41,369 bp for the I45, I69 and NL895 clones, respectively.

The contigs covering the LSC + IRa + SSC region and overlapping the IRb region were further merged into three super contigs of 145,764, 145,929 and 156,380 bp for the I45, I69 and NL895 clones, respectively. The three resulting super contigs for I45, I69 and NL895 represented regions of 1–146,502, 1–146,504 and 1–157,033 bp, respectively, on the *P. trichocarpa* chloroplast genome.

Nevertheless, an approximate 11 kbp of partial sequences on the IRb region was lost in the chloroplast assemblies for both I45 and I69 but was present in those of NL895. IRa and IRb on NL895 were a pair of reverse complementary sequences of 27,649 bp in length and nearly 100% sequence identity, like the IRa and IRb from the *P. trichocarpa* cpDNA. The IRa sequence for NL895 was the same as that of both I45 and I69. In addition, the partial sequence of the IRb region in the I45 and I69 cpDNA was the complete reverse complement of its counterpart in the IRa region. Because the sequences of both IRa and IRb on the *Populus* chloroplast genome formed a pair of inverted repeats that were extremely similar or even identical, it was possible that the IRa and IRb sequences of the clones I45 and I69 were a pair of identical reverse complementary repeats, like those of NL895 and *P. trichocarpa* cpDNA. Using that assumption, the partial sequence lost in the IRb region was inferred from the complete IRa sequence of I45 and I69. Final, we obtained complete chloroplast genome sequences of 156,295 and 156,458 bp for the clones I45 and I69.

### Comparison of the chloroplast genomes

We compared the chloroplast genomes among the three clones (I45, I69 and NL895) in two different ways, comparing their genome sequences and aligning the reads to reference cpDNAs of *P. trichocarpa*. A whole-genome sequence comparison is the most direct way to identify differences in sequences between individuals or genotypes. However, it is very difficult to discover heterozygous loci within an individual using only the sequence comparison. Read mapping to call single nucleotide polymorphisms (SNPs) and insertions/deletions (InDels) complements the whole-genome sequence comparison in discovering differences among the chloroplast genomes of individuals.

#### Comparing whole chloroplast genome sequences

The chloroplast genome sequences of three poplar clones, I45, I69 and NL895, were compared separately with those of *P. trichocarpa* using ‘nucmer’ and ‘show-snps’ in the MUMmer package. A total of 407 variants were identified in the chloroplast genomes of I45, I69 and NL895 compared with the reference cpDNA. These variants consisted of 211 SNPs and 196 InDels. The vast majority of SNP loci (181) were located in the LSC and SSC regions of the cpDNAs, but only 30 SNPs occurred in the IRa and IRb regions. Similarly, only six deletions and six insertions were situated in the IR pair in the chloroplast genome.

Nearly three-quarters of all variants (310) identified in the poplar chloroplast genome were the same across all three poplar clones. The variants consisted of 180 SNPs and 130 InDels, including 67 insertions and 63 deletions. The 180 SNPs were divided into 150 loci in the LSC and SSC regions and 30 loci in the IRa and IRb regions. Only 12 of the 130 InDels were consistent among the three poplar clones and located in the pair of IR regions of the cpDNAs.

The remaining one-quarter of variants (97) in the poplar chloroplast genome were not completely consistent among the I45, I69 and NL895 clones. All of the variants that were inconsistent among I45, I69 and NL895 were located in the LSC (93) and SSC (4) regions, but not in the IRa and IRb regions. The 93 inconsistent variants situated in the LSC region were composed of 30 SNPs, 17 insertions and 46 deletions. All of the inconsistent variants were divided into three groups, including 40 variants that were the same between the two poplar clones I45 and I69 but not to NL895 (marked as I45 = I69), 44 variants that were consistent between I45 and NL895 (I45 = NL895) and 13 variants that were consistent between I69 and NL895 (I69 = NL895). These results seem to suggest that the differences between I69 and I45 chloroplast genomes were less than those between I69 and NL895. This could be because *P. × euramericana* cv. I45 was derived from the cross between *P. deltoides* as the maternal parent and *P. nigra* as the paternal parent. Thus, *P. × euramericana* cv. I45 obtained a *P. deltoides* chloroplast genome, which was very similar to the chloroplast genome of *P. deltoides* cv. I69.

#### Comparing the SNPs obtained by mapping

The SNPs for each dataset of the three poplar clones I45, I69 and NL895 were called by read alignment to the *P. trichocarpa* chloroplast genome using BWA and SAMtools. We used the read mapping strategy to identify 311 variant loci, 71 of which were problematic and discarded. Of these, 68 (> 95.7%) had poor repeatability within the same poplar clone. The other three problematic variants were inconsistent loci at which the predicted reference alleles of the three poplar clones were different. These problematic variants were mainly detected in or near the cpDNA locations containing low-complexity sequences and simple sequence repeats. The vast majority of these problematic loci were consistent with the results obtained by the genome comparison strategy as described previously. Thus, the genome comparison strategy could be used to correct the problematic variants that were identified through the read mapping strategy. In addition, the other 240 variants identified by the read mapping strategy were retained for further analysis.

#### Differences in the variants among the three clones

To discover accurately the differences in the chloroplast genome sequences among the poplar clones I-45, I-69 and NL895, we integrated 407 and 240 variants identified by the chloroplast genome comparison and read mapping strategies, respectively, and obtained 401 variants consisting of 213 SNPs, 108 deletions and 80 insertions. In total, 299 variant loci, approximately three-quarters of all the loci, were located in the LSC region of the poplar chloroplast genome. In addition, the problematic variants, which had been mostly identified using the read mapping strategy, were in low-complexity regions, large InDel regions of > 5 bp in length and short tandem repeat regions (especially the homopolymers polyA or polyT). Six heterogeneous variants, which could not be detected by the genome comparison strategy, were improperly identified using the mapping strategy. Similarly, single-nucleotide variant heteroplasmy had been found in the mitochondrion of mice and human, using Single-Mitochondrion sequencing [11].

All 401 variants were divided into two groups (Additional file 1: Table S5), including 307 variant loci in which the genotypes of the three poplar clones (NL895, I-45 and I69) were identical. The remaining 94 loci with genotypes were not identical among these three clones. Of the 307 identical variants, 42 were situated in the IR pair (IRa and IRb) of the poplar chloroplast genome. None of the variant loci with different genotypes were located in the IRa and IRb regions. Thus, the pair of long interspersed IRs (IRa and IRb) was more conservative than the other two single-copy regions (LSC and SSC), which was consistent with the results found in rice, maize and bamboo [12–14]. This may be due in part to the biased GC content. The overall GC content of the IRa and IRb (41.97%) regions in the poplar cpDNAs was more than those of the LSC (34.47%) and SSC (30.54%) regions.

Of the 307 variant loci having the same genotype among the three poplar clones, most were located in the non-coding regions, such as introns, and upstream and downstream sequences, of the poplar chloroplast genome. Only 65 loci with identical genotypes, 30 silent (synonymous) and 35 missense mutations, were located in the coding regions or exons of the genes. For example, nine of these loci were located in the exon of the RNA polymerase (rpo) gene family, consisting of *rpoA*, *rpoB*, *rpoC1* and *rpoC2*, in the poplar chloroplast genome. The nine loci included a missense mutation in the *rpoA* gene, four silent (synonymous) mutations in *rpoB*, two silent (synonymous) mutations in *rpoC1*, and two missense mutations in *rpoC2*. The α, β, β’ and β” subunits of PEP, which is a key enzyme involved in plant photosynthesis, were encoded by four genes, *rpoA*, *rpoB*, *rpoC1* and *rpoC2* [15]. The vast majority of the other 94 variations with differences in genotypes between the three poplar clones were located in the non-coding region of the poplar chloroplast genome. Nevertheless, only one variant was situated in the coding region of the poplar cpDNA, leading to changes in the amino acid sequence of the PEP β’ subunit encoded by *rpoC1*. The identified genetic variants on exons of *rpoA*, *rpoB*, *rpoC1* and *rpoC2* may have an impact on the transcriptional level of photosynthesis-related genes, which are mediated by PEP [3]. The deletion of each of *rpoA*, *rpoB*, *rpoC1* and *rpoC2* can cause defects in plant photosynthesis [15, 16]. However, no photosynthetic defects were found in the three poplar clones (I45, I69 and NL895). Thus, the identified genetic variants on exons of *rpoA*, *rpoB*, *rpoC1* and *rpoC2* of I45, I69 and NL895 should not cause loss or gain of these *rpo* genes functions.

The genotypes of the loci identified by genome sequence comparison strategy were consistent with those identified by the read mapping strategy. The genotypes of the loci of the poplar clone NL895 were identical to those of its maternal parental clone, I69, and different from those of its male parental clone, I45. Thus, the offspring cpDNAs were derived from the female parent in species of the genus *Populus*.

## Discussion

### Chloroplast genome construction

In the study, we constructed high-quality chloroplast genomes for three poplar clones, the superior clone NL895 and its female (I69) and male (I45) parents, from 15 to 16× whole-genome shotgun sequences per clone on the HiSeq 2000 platform using a combination of de novo and reference-assisted strategies. A high-quality assembly of the chloroplast genome is essential for comparing chloroplast genomes between closely related species or individuals, especially for genetic relationships between parent and offspring or between full-sibs. However, chloroplast genome assemblies are challenging due to influences of both sequencing design and assembly strategies and the degree of kinship between the reference and species to be assembled.

#### Sequencing design

The length, number and distribution of short reads used for assembling cpDNA are the three primary features of sequencing design. The length of the reads generated on Illumina sequencing platforms appeared to not affect the assembly of the chloroplast genomes for the three poplar clones, and this was supported by the resulting assemblies of both the simulated Illumina-like reads and the real Illumina reads. However, this only indicates that there are no significant differences in the de novo assembly among Illumina reads ranging from 60 to 120 bp in length. Compared with the short reads of the Illumina sequencing platform, the longer reads of the third generation sequencing platform (such as PacBio long reads having a mean length of > 10 kb, http://www.pacb.com/) have the potential to resolve long repeats in the chloroplast genome and make it relatively easy to de novo assemble the chloroplast genome. For example, the complete chloroplast genome of pineapple (*Ananas comosus*) containing a pair of IR regions (IRa and IRb) 26.7 kbp in size has been constructed using PacBio long reads [17]. However, a high error rate (~ 15%) and relatively high cost per base pair are two shortcoming of PacBio long reads compared with Illumina short reads [18]. Thus, the hybrid assembly of Illumina short reads with their high accuracy and PacBio long reads with their low accuracy may be a promising strategy for constructing high-quality chloroplast genomes.

The number of reads used for a single independent assembly of a chloroplast genome is a factor that causes differences in the resulting assemblies. The assemblies based on simulated and real datasets in this study did not suggest that more reads result in superior assemblies. A million pairs of chloroplast reads (10^6^) is a suitable amount for the de novo assembly of poplar cpDNAs. However, the suitable number of whole-genome shotgun sequencing reads for assembling cpDNAs depends on the ratio of chloroplast reads in the whole-genome sequencing data as well as the number of chloroplast reads to be assembled. There are differences in the chloroplast ratios of whole-genome sequencing reads among organisms or clones. The cpDNA ratios of the three poplar clones, NL895, I45 and I69, were 5.51, 4.99 and 8.82%, respectively. The cpDNA ratios of the Sanger sequencing data for two rice cultivars (PA64S and 93–11) are less than 2.3% [12]. Thus, it is necessary to determine the whole-genome reads amount to estimate the cpDNA ratio in advance of whole-genome shotgun sequencing.

The uneven coverage distribution of reads is an inherent defect of second-generation sequencing platforms (such as Illumina sequencing platforms), just like the distribution of Illumina HiSeq reads in this study [19]. The read depths of gaps or missing regions compared with the reference cpDNA is usually the lowest peak and an obstacle for the complete cpDNA assembly. In contrast, the PacBio sequencing platform is able to generate a uniform distribution of reads across the entire genome [20].

Although the PacBio sequencing platform has many advantages, such as longer reads and uniform coverage distributions, second-generation sequencing platforms (such as Illumina platforms) remain proper platforms for de novo cpDNA assembly owing to the accuracy, extremely low single-nucleotide cost, high reproducibility and high-throughput of reads from these platforms [21]. Additionally, multi-pass sequencing, to some extent, can remedy the deficiency of short Illumina reads. In particular, when high-quality cpDNA of closely-related species are available, the Illumina sequencing platform is a relatively good choice for simultaneously assembling cpDNA of multiple samples from organisms that are closely related (such as parent–offspring and full-sibs) and identifying mutations in their cpDNAs.

#### Assembly strategy

De novo assembly and contig merging are the two main steps for cpDNA construction in this study. Eight de novo assemblers, Velvet, SOAPdenovo2, ABySS, Minia, Edena, SGA, IDBA and SPAdes, were used for the de novo-assembly of all 47 datasets from the three poplar clones (NL895 and its parents I45 and I69). Compared with the other five assemblers, ABySS, IDBA and SPAdes provided relatively superior results in terms of the metrics used in the assessment of chloroplast genome assemblies. The resulting assemblies generated using the other five assemblers had some deficiencies, such a large numbers of fairly short contigs. In addition, the parameter k-mer specified in the four assemblers, ABySS, Minia, SOAPdenovo2 and Velvet, influenced the de novo chloroplast assemblies.

It is necessary to order and orient the de novo-assembled contigs. We applied the contig integrator CISA to merge de novo assemblies of multiple datasets for each of the clones. The quality levels of these cpDNAs were slightly improved, particularly in the accuracy and continuity of the resulting assemblies.

#### Kinship

High-quality chloroplast genomes are very important for comparing chloroplast genomes and accurately identifying mutations in cpDNAs, specifically for parents–offspring or F_1_ full-sib progeny comparisons. The genetic distance between the target genome to be assembled and its reference genome is an important factor for the de novo assembly of chloroplast reads isolated from whole-genome sequencing data. For example, it is difficult to capture cpDNA reads in regions that are inserted or deleted in the target cpDNA compared with in the reference chloroplast genome. In this study, we chose the *P. trichocarpa* cpDNA as a reference to extract almost all of the chloroplast reads from the whole-genome shotgun reads of the three clones. Nevertheless, the construction of the chloroplast genomes for the three poplar clones revealed that it was still difficult to construct high-quality poplar chloroplast genomes using only the isolated cpDNA reads from single-pass sequencing data. This may be due in part to the 27,649-bp IRa and IRb sequences with nearly 100% sequence identity. Finally, we obtained high-quality and nearly-complete chloroplast genome sequences for the three poplar clones by merging contigs of multiple assemblies per poplar clone.

### Chloroplast variations in the transmission from one generation to the next

In the study, we used an entire chloroplast genome comparison to preliminarily determine the pattern of the spontaneous mutations in the chloroplast genome of a poplar hybrid F_1_ generation. During the transmission of poplar chloroplasts from female parent to offspring, the chloroplast’s mutations were mainly located in the non-coding and single-copy regions. This may help to explain differences in breeding effects between a cross and its reciprocal cross. These results contribute important auxiliary information for forestry tree breeding, such as parental selection and the prediction of potential mutations based on chloroplast genomes of female and male parents. However, spontaneous mutations in full-sib families have rarely been reported, such as in *Drosophila melanogaster* [22]. Thus, the identification of spontaneous mutations in a full-sib family using whole chloroplast genome comparisons is a prerequisite for breeding programs.

Additionally, we identified the female parent of a natural hybrid clone as I45 by comparing cpDNAs of I45 and *P. deltoides* clone I69, which suggests that a whole chloroplast genome comparison can be used to determine the female origins of varieties and clones. Information on the parental origins of hybrid varieties are important for poplar evolutionary studies and breeding programs.

### Future studies

While this research is insufficient to completely illustrate that the law of genetic variation occurred in the chloroplast genomes within a *Populus* full-sib family, it builds a framework for studying and understanding the relationships of spontaneous *Populus* chloroplast mutations with its growth traits. Thus, in future studies, we will construct the chloroplast genomes of another 64 progeny from the same full-sib family using the same strategy and the three high-quality cpDNA sequences generated here. Additionally, we will perform association analyses of poplar chloroplast genotypes in combination with 24-year growth-associated phenotypic data for two parents and 64 of their offspring.

## Methods

### Chloroplast genome resources and computing environment

The complete chloroplast genome sequences of six poplar species, *P. alba* (GenBank accession no. NC_008235), *P. tremula* (NC_027425), *P. euphratica* (NC_024747), *P. fremontii* (NC_024734), *P. balsamifera* (NC_024735) and *P. trichocarpa* (NC_009143), were downloaded from Organelle Genome Resources at NCBI (http://www.ncbi.nlm.nih.gov/genome/organelle/). The six poplar species belong to four sections of the genus *Populus*, sect. *Populus* (*P. alba* and *P. tremula*), sect. *Turanga* (*P. euphratica*), sect. *Aigeiros* (*P. fremontii*) and sect. *Tacamahaca* (*P. balsamifera* and *P. trichocarpa*).

The analysis and mining of large-scale sequence datasets, such as read alignment, SNP calling and sequence assembly, was conducted on a DELL PowerEdge R910 server with 512 GB RAM (32 × 16 GB). Ubuntu is the Linux operating system on the R910 server.

### Pre-assessing reference-assisted strategy

To select the right reference from the six poplar cpDNAs, two steps were performed. Firstly, to estimate the sequence similarities between these cpDNAs, pairwise genome alignments were conducted using LASTZ (v1.03.28, http://www.bx.psu.edu/~rsharris/lastz/) with at least a 95% identity. Secondly, to infer the efficiency of each poplar cpDNA as a reference genome, the number of the identified cpDNA reads was calculated based on the alignment of the simulated short reads against each poplar cpDNA. The split-reads method, which split the reference sequence into many 100-bp fragments with a 1-bp sliding window, was used to generate simulated short reads from these poplar cpDNAs. Mapping the short reads against cpDNAs for each of the six *Populus* species was performed using BWA (v0.7.12) and Bowtie (v1.1.1) with default parameters [23, 24].

To evaluate the feasibility of the reference-assisted strategy for the poplar chloroplast genome assembly, we utilized the reads simulator wgsim (v0.3.0, https://github.com/lh3/wgsim) to simulate and generate Illumina-like paired-end reads in an attempt to assemble poplar cpDNA from Illumina-like reads. Based on the above findings, the *P. trichocarpa* chloroplast genome (NC_009143) was chosen as a reference sequence for the wgsim simulator. The duplicates of the simulated paired reads were identified and discarded with FastUniq (v1.1) [25]. The genome size for the Illumina-like reads generated by wgsim was predicted using KmerGenie (v1.6982) [26]. The simulated reads were de novo assembled into the cpDNA by Minia (v2.0.3) [27]. These Minia assemblies were assessed using the assembly evaluation tool QAUST (v3.1) with *P. trichocarpa* cpDNA as a reference genome [28].

### Sampling and sequencing

The *Populus* materials used in the study were harvested from the Zhangji Forestry Centre at Xuzhou, Jiangsu Provinces, China. DNA samples were taken from the fresh green leaves of three *Populus* sect. *Aigeiros* clones, I69 (*P. deltoides* cv. ‘I-69/55’), I45 (*P. × euramericana* cv. ‘I-45/51’), and NL895, which is a genotype from the F_1_ progeny of the interspecific hybrids between I69 (female parent) and I45 (male parent). Total DNA was extracted from the fresh leaves for each *Populus* clone using a DNeasy Plant Mini Kit (QIAGEN, Hilden, Germany, Cat No.69104) according to the manufacturer’s instructions. The quantification and purity of the extracted DNA was assessed using agarose gel electrophoresis and a NanoDrop 2000 spectrophotometer. The extracted DNA was used for paired-end sequencing on the Illumina HiSeq 2000. DNA sequencing was carried out 15–16 times for each clone. The whole-genome shotgun sequencing of the three *Populus* clones was performed at the Chinese National Human Genome Center at Shanghai (http://www.chgc.sh.cn/).

### Extracting cpDNA reads

A quality assessment of the raw short reads generated on Illumina HiSeq platform were carried out using FastQC (v0.11.5, http://www.bioinformatics.babraham.ac.uk/projects/fastqc/), and quality control measures were performed with the FASTX-Toolkit (v0.0.14, http://hannonlab.cshl.edu/fastx_toolkit/). The low-quality ends were trimmed from the raw short reads using the FASTX-Toolkit. The trimmed reads were used for further analyses.

The cpDNA reads were isolated from whole-genome shotgun read data by mapping the trimmed reads to the reference chloroplast genome. The trimmed reads were mapped onto the *P. trichocarpa* chloroplast genome (NC_009143) using Bowtie and BWA. The paired-end reads mapped onto the *P. trichocarpa* chloroplast genome using both Bowtie and BWA were screened and integrated as the cpDNA reads for the chloroplast genome assembly of the three poplar clones. The duplicates were identified from the paired-end reads of poplar chloroplast using FastUniq (v1.1) [25] and discarded.

### Constructing chloroplast genomes

Frist, a de novo assembly strategy was employed to make the initial assembly of the selected cpDNA reads. The cpDNA reads were assembled into the initial contigs for each dataset from the three clones using eight de novo genome assemblers, Velvet (v1.2.10), SOAPdenovo2 (v2.04), ABySS (v1.9.0), Minia (v2.0.3), Edena (v3.131028), SGA (v0.10.13), IDBA (v1.1.1) and SPAdes (v3.6.0) [27, 29–35]. These eight de novo assemblers were grouped into four parameter-based types: (I) ABySS, Minia, SOAPdenovo2 and Velvet were used with 23 odd k-mer values from 19 to 63 for poplar cpDNA assembly; (II) SGA was applied with six various overlapping sizes (−m) of 41, 45 51, 55, 61 and 63; (III) IDBA was utilized with six different iterative step values (−step) of 2, 4, 8, 10, 20 and 30; (IV) Edena and SPAdes were run under their respective default parameters only. A total of 4982 de novo assemblies were performed on all 47 read datasets from the three poplar clones in the study. In addition, the running-time for each assembly was recorded by an in-house Perl script.

Subsequently, the resulting assemblies for the 47 datasets were assessed using QUAST with the *P. trichocarpa* cpDNA (NC_009143) as a reference [28]. A visual inspection of the cpDNA assemblies was conducted on the integrative genomics viewer IGV (v2.3.67) [36].

To order and orient the contigs assembled from multiple datasets for each clone, we utilized the contig integrator CISA (v1.3) to merge contigs from various assemblies for the same clone, and these were manually curated into a contig covering the LSC + IRa + SSC region of the chloroplast genome [10]. Then, the de novo assembled contigs over the IRb region were screened based on the QUAST assessments and merged into a contig by CISA. Contigs spanning both the LSC + IRa + SSC and IRb regions were manually merged into contigs based on the reference cpDNA.

The quality of the constructed cpDNAs for the three clones was assessed by QUAST with the *P. trichocarpa* cpDNA as the reference. The depth of reads across the entire cpDNA per dataset was calculated from the BAM format using ‘genomecov’ in the bedtools package (v2.25.0) [37]. Gaps in assemblies for the same clone were combined by ‘merge’ in the bedtools package.

### Comparing chloroplast genomes

The constructed chloroplast genomes for the three poplar clones were annotated with DOGMA (http://dogma.ccbb.utexas.edu/) [38]. BLAST algorithm-based searches against annotated genes from six poplar reference cpDNAs were performed to detect potential genes in the cpDNAs of the three clones. tRNA genes in these cpDNAs were identified by tRNAScan-SE (v1.21, http://lowelab.ucsc.edu/tRNAscan-SE/) [39].

The chloroplast genomes for the three poplar clones, including NL895 and its parents I69 and I45, were pairwise compared using MUMmer (v3.23, http://mummer.sourceforge.net/) [40]. Four regions, LSC, IRa, SSC and IRb, were identified by ‘repeat-mach’ in the MUMmer package.

The reads per dataset were aligned to the corresponding cpDNA assembled in the study using BWA (v0.7.12). SNPs and InDels (Insertions and Deletions) were detected directly from each of the 47 datasets by SAMtools (v1.2) and BCFtools (v1.2) [41, 42]. The annotation of the called variant loci was performed using SnpEff with the *P. trichocarpa* cpDNA as the reference.

## Conclusions

In this study, the high-quality and complete chloroplast genomes for three poplar clones, the superior clone NL895 and its both parents I69 (*P. deltoides* Bartr. cv. ‘I-69/55’, ♀) and I45 (*P. × euramericana* Guinier. cv. ‘I-45/51’, ♂), were constructed using ultra-depth coverage (>8500×) of chloroplast genome per clone. The three high-quality chloroplast genomes can serve as an important resource for further studying the chloroplast variation pattern within full-sib family of I69 and I45. Furthermore, the chloroplast spontaneous mutations between parents and offspring provide a potential application of cpDNA information in *Populus* breeding via molecular design.

## Additional file

Additional file 1: **Table S1.** The ratio of split-reads mapped to each cpDNA of six *Populus* species using Bowtie and BWA. **Table S2.** The genome size of *P. trichocarpa* cpDNA was estimated using each of twelve sets of simulated reads (60 bp–10 k, 60 bp–100 k, 60 bp–1 M, 60 bp–10 M, 80 bp–10 k, 80 bp–100 k, 80 bp–1 M, 80 bp–10 M, 100 bp–10 k, 100 bp–100 k, 100 bp–1 M and 100 bp–10 M). **Table S3.** Basic statistics of 13 assemblies used for further analyses. **Table S4.** The cpDNA and gDNA ratios of read datasets for the three poplar clones I45, I69 and NL895. **Table S5.** The positions, reference and alternative base, types (Replace|Insert|Delete) and the genotypes of all 401 variants identified in the three poplar clones. (XLSX 31 kb)

## Abbreviations

ANOVA: Analysis of variance
cpDNAs: Chloroplast DNAs
InDels: Insertions/Deletions
IRs: Inverted repeats
kbp: Kilobase pairs
LSC: Large single-copy
NGS: Next-generation sequencing
PEP: Plastid-encoded RNA polymerase
RuBisCO: Ribulose-(1,5)-bisphosphate carboxylase/oxygenase
sib: Sibling
SNPs: Single nucleotide polymorphisms
SSC: Small single-copy
